# Perceptual advantage for category-relevant perceptual dimensions: the case of shape and motion

**DOI:** 10.3389/fpsyg.2014.01394

**Published:** 2014-12-01

**Authors:** Jonathan R. Folstein, Thomas J. Palmeri, Isabel Gauthier

**Affiliations:** ^1^Department of Psychology, Florida State UniversityTallahassee, FL, USA; ^2^Psychological Sciences, Vanderbilt UniversityNashville, TN, USA

**Keywords:** category learning, perceptual learning, psychophysics, object recognition, dimensional modulation

## Abstract

Category learning facilitates perception along relevant stimulus dimensions, even when tested in a discrimination task that does not require categorization. While this general phenomenon has been demonstrated previously, perceptual facilitation along dimensions has been documented by measuring different specific phenomena in different studies using different kinds of objects. Across several object domains, there is support for *acquired distinctiveness*, the stretching of a perceptual dimension relevant to learned categories. Studies using faces and studies using simple separable visual dimensions have also found evidence of *acquired equivalence*, the shrinking of a perceptual dimension irrelevant to learned categories, and *categorical perception*, the local stretching across the category boundary. These later two effects are rarely observed with complex non-face objects. Failures to find these effects with complex non-face objects may have been because the dimensions tested previously were perceptually integrated. Here we tested effects of category learning with non-face objects categorized along dimensions that have been found to be processed by different areas of the brain, shape and motion. While we replicated acquired distinctiveness, we found no evidence for acquired equivalence or categorical perception.

## Introduction

**Figure 1:**
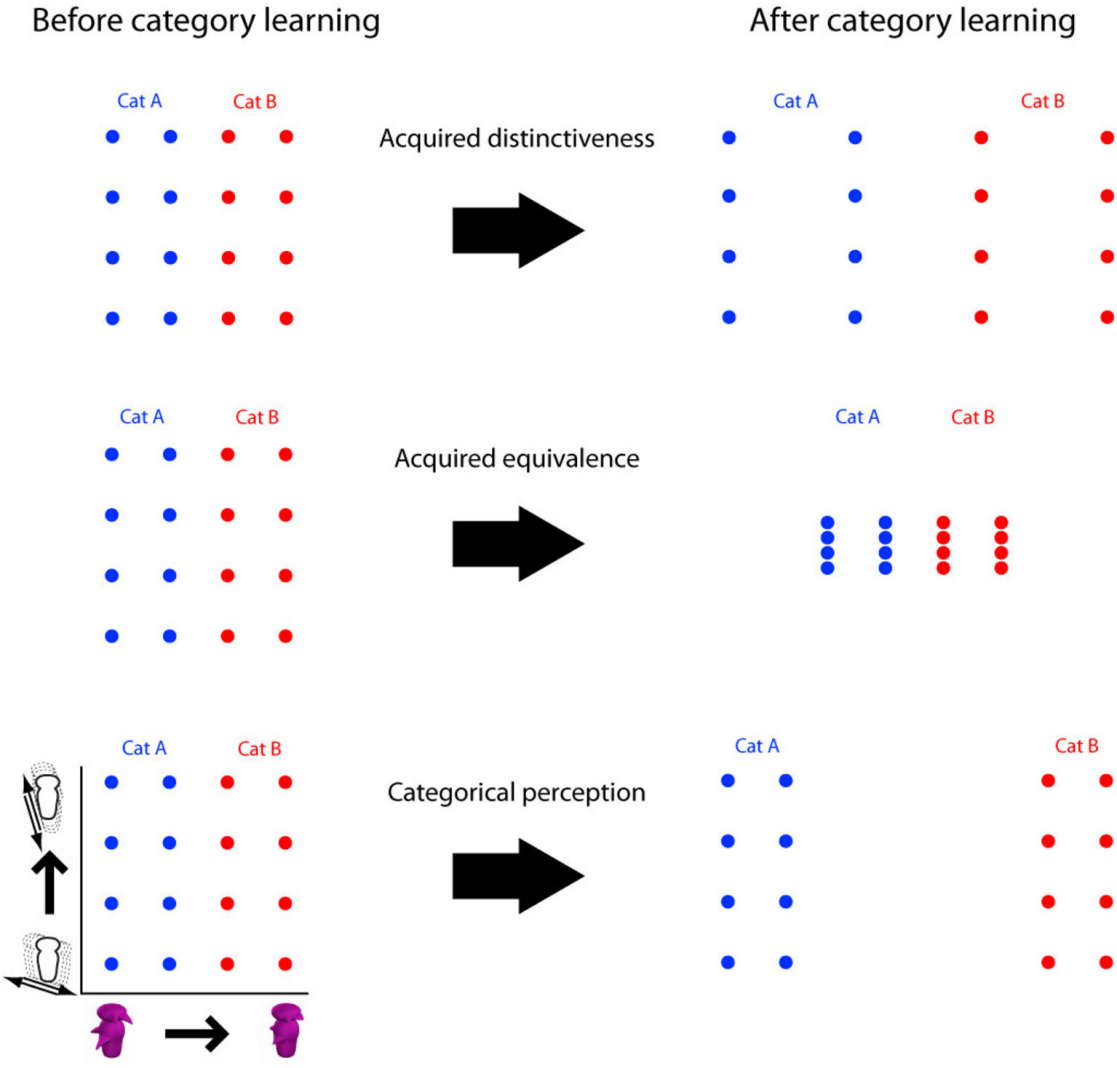
**Three types of dimensional modulation as a consequence of category learning**. Dots represent positions of stimuli within a 2-dimensional space of objects. Changes in discriminability are represented by stretching and shrinking of the space. Acquired distinctiveness: a global increase in discriminability along the category relevant dimension. Acquired equivalence: a decrease in discriminability along the irrelevant dimension. Categorical perception: an increase in discriminability local to the region around the category boundary.

To recognize, categorize, or name objects, many theories propose that objects are first represented in terms of their perceptual features and dimensions. There is growing evidence that category learning alters these perceptual features and dimensions in important ways, forming a family of phenomena referred to as *dimensional modulation* (Figure 1). In the most common case of *acquired distinctiveness*, category learning causes a general perceptual advantage for object dimensions that are relevant for categorization compared to irrelevant dimensions. For instance, if objects vary in size and brightness, learning to categorize those objects according to size results in an increase in ability to visually discriminate two objects with small differences in size (Goldstone, 1994).

Acquired distinctiveness has been observed using stimuli composed of a range of perceptual dimensions, including dimensions that define object shape. For instance, it is observed using stimuli that vary orthogonally along the psychologically separable dimensions of curvature and aspect ratio (Op de Beeck et al., 2003; De Baene et al., 2008). Acquired distinctiveness is also observed using complex stimuli defined far more abstractly, for example by *morphing* faces or other complex objects. In one approach, a two-dimensional space of morphed objects is created, with one dimension defined by the morph between one pair of objects (a morphline) and the other dimension defined by a second pair of objects (another morphline). These two morphlines are then used as two orthogonal dimensions to define a two-dimensional space of complex objects. The objects at positions along these morphlines are then morphed factorially to fill the space (Goldstone and Steyvers, 2001; Folstein et al., 2012a). Participants can learn to categorize this space according to a boundary that divides the space orthogonal to one of the dimensions, making that dimension relevant, and parallel to the other dimension, making that dimension irrelevant. Analogously to the case of size and brightness discussed at the outset, acquired distinctiveness is observed when stimuli that vary along the relevant morph dimension are better discriminated as a consequence of having learned to categorize them along the relevant dimension.

The broader phenomenon of dimensional modulation, which includes acquired distinctiveness, exhibits several key features, some of which are observed more consistently than others: First, it can be *task-independent*, in the sense that changes in visually discriminability as a consequence of category learning can be observed in tasks that do not require categorization^1^. For example, following category learning, enhanced visual discriminability has been measured using a pairwise same-different task. Even though any dimension may be relevant in the same-different discrimination of pairs of objects, the dimension that was relevant during category learning retains a perceptual advantage (Goldstone, 1994; Folstein et al., 2012a). It is as if a dimension relevant to the previously learned category has been “stretched” relative to the irrelevant dimensions. This perceptual advantage remains unchanged even when no intervening category learning has been performed for several days (Folstein et al., 2012b). And a task-independent neural stretching commensurate with the perceptual stretching has been observed in ventral temporal cortex (Folstein et al., 2013).

Second, in addition to enhanced perceptual discriminability along the category-relevant dimension (acquired distinctiveness), some studies have also observed decreased perceptual discriminability along category-irrelevant dimensions (*acquired equivalence*). Acquired equivalence has been observed using face morph dimensions and relatively simple dimensions of size and brightness using square patches (Goldstone, 1994; Goldstone and Steyvers, 2001; Goldstone et al., 2001). Other studies using complex objects have found no evidence for acquired equivalence (Op de Beeck et al., 2003; Folstein et al., 2012a; Van Gulick and Gauthier, 2014).

Third, while increases in discriminability from category learning are often observed along the entire relevant dimension, there has been mixed evidence for an additional selective advantage for regions of perceptual space spanning the category boundary, the classic phenomenon of *categorical perception* (Harnad, 1987)^2^. Perhaps the best evidence for category-learning-induced categorical perception in the visual domains comes again from studies using morphed faces (Gureckis and Goldstone, 2008) and from studies using objects defined by simple psychophysical dimensions (Goldstone, 1994; Özgen and Davies, 2002; Notman et al., 2005); one recent study found categorical perception for single-dimensional morphlines created by blending exactly two objects (Wallraven et al., 2014). By contrast, other studies have not found categorical perception effects; these often used complex multi-dimensional object spaces defining object shape, such as morphspaces created by blending four or more objects and object spaces defined by separable dimensions like curvature and aspect ratio (Op de Beeck et al., 2003; Jiang et al., 2007; Folstein et al., 2012a, 2013; Van Gulick and Gauthier, 2014). So, although acquired distinctiveness along category-relevant dimensions is commonly observed, acquired equivalence and categorical perception are not. The latter have been observed for faces and simple dimensions but not for multidimensional spaces defining complex non-face objects.

**Figure 2:**
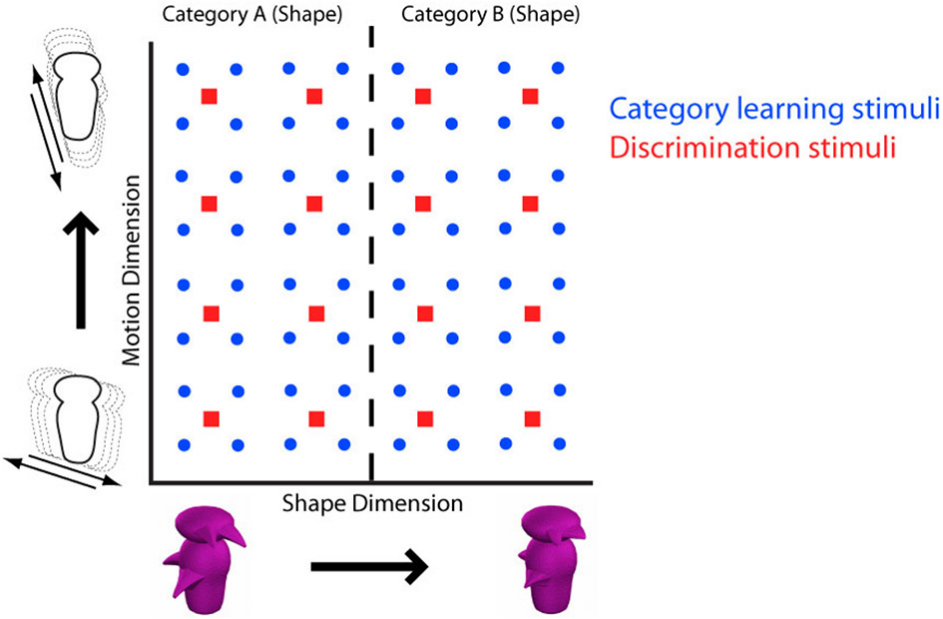
**Stimulus space defined by dimensions of shape (horizontal axis) and motion (vertical axis)**. Red squares represent stimulus positions within the 4 × 4 space, blue circles represent positions within the 8 × 8 space. Categorization according to shape is illustrated by the dashed line, making shape the relevant dimension. When participants learned to categorize according to motion, motion became the relevant dimension.

There are many methodological differences between the various studies reviewed above. Studies that have failed to find evidence for acquired equivalence and categorical perception using non-face objects have often used multidimensional object spaces defined using morphing techniques. The result is a complex multidimensional shape space with constituent dimensions that often defy any intuitive description. Here, we instead test for various forms of dimensional modulation produced by category learning using stimulus spaces of complex objects defined by two distinct dimensions: shape and motion (Figure 2).

Shape and motion dimensions have properties that could align themselves with previous studies that have reported acquired equivalence and categorical perception. In our experiment, the shape dimension is a single morphline between two novel Greeble objects, which is similar to the single morphine used by Wallraven et al. (2014), who observed categorical perception. The motion dimension consists of the angle with which the Greeble sweeps back and forth; while we know of no tests of separability in the Garner (1974) sense between object shape and rigid motion, the fact that these two dimensions are represented in separate and distinct brain areas suggest that the dimensions may be separable (e.g., Li et al., 2007). So in much the same way that dimensions of size and brightness have been shown to be separable and produce categorical perception, object shape and motion could be positioned to do the same (Garner, 1974; Goldstone, 1994).

In fact, we will show that despite these apparent similarities to stimuli used by past studies reporting acquired equivalence and categorical perception, we find the same cluster of dimensional modulation effects we have found in past work with complex objects: general acquired distinctiveness after category learning for the category-relevant dimension but neither acquired equivalence nor categorical perception.

## Procedure

### Participants

Twenty-two students from the Vanderbilt community participated in the experiment over two sessions. Twelve learned to categorize the stimulus space according to the motion dimension and 10 learned to categorize it according to the shape dimension. Four additional subjects were dropped because categorization accuracy was less than 55% during one or both training sessions. Participants received $12 per h for their participation.

### Stimuli

The stimulus set was created by a factorial combination along the perceptual dimensions of shape and motion (Figure 2). The shape dimension was created by morphing two artificial Greeble objects (asymmetrical versions of the Greebles originally used by Gauthier and Tarr, 1997). The motion dimension was created by having the Greebles move from side to side at angles varying between 18° and 72° off of vertical. A 4 × 4 space with four evenly spaced motion angles (see Supplemental Material) by four evenly spaced proportions along the morphline was used in the discrimination task and a similarly constructed 8 × 8 space (with no overlap with the 4 × 4 space) was used in the category learning task (see Folstein et al., 2013 for a stimulus spaces with a similar structures^3^).

### Procedure

Participants performed a same-different discrimination pre-test, followed by two sessions of category learning, followed by a same-different discrimination post-test. During the category learning sessions, separate participant groups learned to categorize the stimuli according to shape or angle of motion. The experiment was conducted in two sessions on two separate days, each lasting approximately 2 h each. The discrimination pretest and the first session of category learning were conducted on the first day and the second session of category learning and discrimination post-test were conducted on the second day.

#### Discrimination task

Discrimination trials consisted of S1, a mask, and S2. Participants were instructed to indicate whether S1 and S2 were identical or (very slightly) different. Specifically, S1 and S2 were either identical or occupied adjacent positions of the 4 × 4 stimulus space, differing along either the shape or motion dimensions. Depending on which dimension defined the learned category, shape or motion were category-relevant or category-irrelevant. Some pairs crossed the middle of the space while other pairs did not, allowing us to evaluate categorical perception effects across the category boundary.

The duration of S1 and S2 was 1.5 s and the duration of the mask was 0.7 s. There were a total 112 identical trials, 168 different-shape trials, and 168 different-motion trials. All adjacent pairs within the space were presented an equal number of times and all possible pairs of identical stimuli were presented an equal number of times.

#### Category learning task

Participants learned to categorize the stimuli according to a category boundary that divided the 8 × 8 space in half along the shape or motion dimension. On each category learning trial, a randomly selected stimulus was presented for 1.5 s followed by a mask for 0.7 s. Participants were allowed to respond as soon as the stimulus appeared. Feedback on accuracy was provided after each response (duration 1 s). The feedback was verbal and indicated the correctness of the response and the correct category (e.g., the words “Correct, Mog” appeared on the screen).

## Results

### Categorization task

Categorization accuracy and reaction time were entered into a 2 × 2 ANOVA with factors of Training and Session. Across the two sessions, categorization accuracy improved from 89 to 91% in the Motion group and from 85 to 89% in the Shape group. Reaction times were also faster in the second session of training. There was a main effect of Session for both accuracy and reaction time [accuracy: *F*_(1, 20)_ = 19.9, *MSe* = 0.001, *p* < 0.005, η^2^_*p*_ = 0.50; reaction time: *F*_(1, 20)_ = 4.4, *MSe* = 0.015, *p* < 0.05, η^2^_*p*_ = 0.18]. There was also a main effect of Training, reflecting higher accuracy in the Motion group than the Shape group [*F*_(1, 20)_ = 5.6, *MSe* = 0.002, *p* < 0.05, η^2^_*p*_ = 0.22]. No other effects were significant. What is clear from these results is that participants learned the categories as instructed. The critical question then is whether this learning had an effect on perceptual discrimination.

### Discrimination task

An initial comparison of discriminability along the shape and motion dimensions during pretest suggested that the two dimensions were equally discriminable [*t*_(21)_ = 1.14, *p* = 0.269].

In the main analysis, sensitivity (d′) was calculated for pairs that differed along the relevant dimension vs. pairs that differed along the irrelevant dimension. The data were entered into a 2 × 2 × 2 × 2 ANOVA with factors of Dimension (motion relevant vs. shape relevant), Relevance (relevant dimension vs. irrelevant dimension), Position (discrimination pairs that crossed middle of the space vs. discrimination pairs on outside of the space) and Test (pre-test vs. post-test).

**Figure 3:**
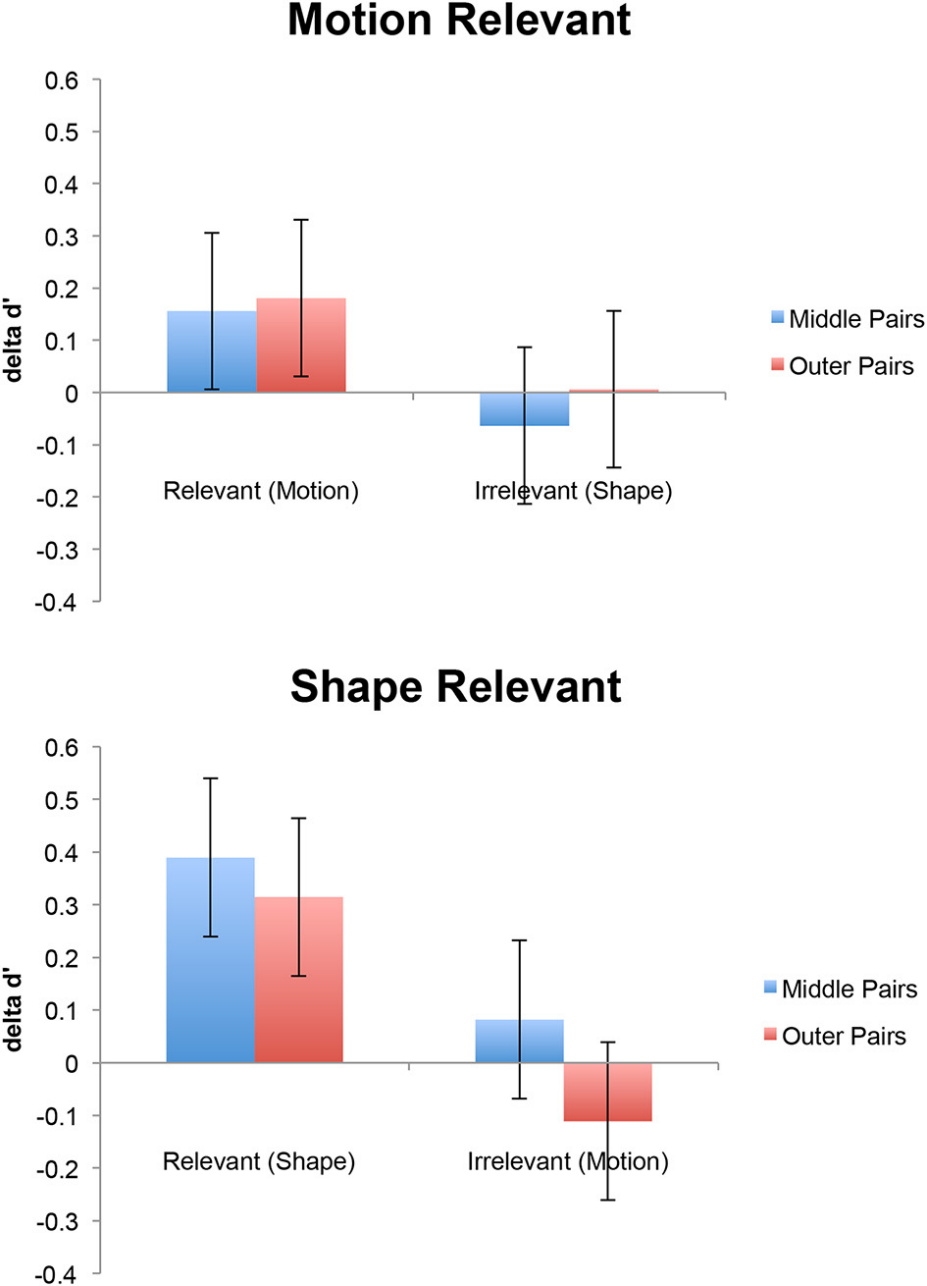
**Improvement in discrimination performance (delta d′) for participants trained to categorize by motion (top panel) or shape (bottom panel)**. Blue bars show pairs that crossed the middle of the space and red bars show pairs that do not. Error bars are 95% confidence intervals for the Relevance × Test interaction (Masson and Loftus, 2003).

The results showed that category learning caused an increase in discriminability along the category-relevant dimension, but no evidence for categorical perception or acquired equivalence (Figure 3). Global acquired distinctiveness was confirmed by a significant two-way interaction between Relevance and Test [*F*_(1, 20)_ = 7.44, *MSe* = 0.12, *p* < 0.03, η^2^_*p*_ = 0.27]. Learned categorical perception would have been apparent in the interaction between Relevance, Test, and Position, but this interaction was not significant [*F*_(1, 20)_ < 1]. Of little theoretical interest, there was also a main effect of Dimension [*F*_(1, 20)_ = 4.4, *MSe* = 0.33, *p* < 0.05, η^2^_*p*_ = 0.18] and a main effect of Position [*F*_(1, 20)_, *MSe* = 0.047, *p* < 0.01, η^2^_*p*_ = 0.39]. The main effect of Dimension reflected generally higher discrimination performance (for both shape and motion) in the participants trained to categorize by motion while the main effect of Position reflected generally higher performance for pairs on the outside of the space vs. those crossing the middle (both before and after category learning); the effect of Position is could be caused by the outer motion positions being closer to the cardinal directions of 90° and 0° or possibly by anchoring effects in which stimuli from extreme parts of a stimulus space are easier to identify (e.g., Luce et al., 1982). The facts that the effect of Position did not change between pre-test and post-test and that category learning had an equal effect on middle pairs and outer pairs (ruling out ceiling and floor effects) suggests that unequal discriminability within the space cannot account for our failure to observe learned categorical perception. Neither of these findings will be discussed further.

Figure 3 shows clearly that category learning increased discriminability for category-relevant dimensions and had little effect on irrelevant dimensions, demonstrating an effect of acquire distinctiveness but not acquired equivalence. This was confirmed by planned *t*-tests. d′ for relevant dimensions improved significantly from pretest to post-test [*t*_(21)_ = 2.7, *p* = 0.01], but d′ for irrelevant dimensions did not change reliably [*t*_(21)_ = −0.42, *p* = 0.68]. An a priori power analysis based on the acquired equivalence effect observed by Goldstone (1994) along the brightness dimension (*d* = 0.727) showed that we needed 17 subjects to achieve a power of 0.8.

We also analyzed the acquired distinctiveness effect separately for the two training groups (shape categorizers and motion categorizers), again comparing d′ for pre-test to d′ for post-test separately along the relevant and irrelevant dimension for each group. While there was a trend toward improvement along the relevant dimension for both groups, neither effect reached significance [Motion: *t*_(11)_ = 1.68, *p* = 0.12, Shape: *t*_(9)_ = 2.04, *p* = 0.071]. Effects along the irrelevant dimension did not approach significance [Motion: *t*_(11)_ = −0.048, Shape: *t*_(9)_ = −0.568].

Finally, because learned categorical perception was of special interest, we separately analyzed the learning effects for relevant pairs that crossed or did not cross the category boundary. First we calculated delta d′ by subtracting pretest d′ from posttest d′ for each pair type and then compared delta d′ for boundary crossing vs. non-boundary crossing relevant pairs. Consistent with our ANOVA, the effect was not significant [*t*_(21)_ = 0.208, *p* = 0.837]. An a priori power analysis based on the categorical perception effects observed by Goldstone (1994) suggested that, to achieve a power of 0.8 with alpha 0.05, we needed 9 subjects based on the effect size along Goldstone's brightness dimension (*d* = 1.11) and 14 subjects based on the effect for the size dimension (*d* = 0.822). This suggests that we had more than sufficient power to detect effects of the size observed by Goldstone (1994). Of course it is possible that much smaller effect sizes could go undetected. With our sample size of 22, we could detect an effect size of *dz* = 0.62 with power of 0.8, and a medium effect size of *dz* = 0.49 with power of 0.6. Small effect sizes of *dz* = 0.2 or below would be well beyond our power to detect. Thus it is possible that we failed to detect a categorical perception effect because the effect size for our space is much smaller than the effect size observed in Goldstone's space of size and brightness. Whether there was no effect at all or a much smaller effect, the question is why.

Results for reaction time were qualitatively similar, but weaker, with no evidence of a speed-accuracy trade-off, and are not reported here.

## Discussion

The purpose of the experiment was to examine the effect of category learning on perceptual discrimination of objects in a multidimensional space defined by shape and motion. Participants learned to categorize objects that varied in shape or in the direction of motion. Category learning caused acquired distinctiveness for objects differing along the category-relevant stimulus dimensions, but did not cause acquired equivalence along category-irrelevant dimensions and did not cause categorical perception. While our ability to make inferences based on those null results is of course limited, it is clear that global acquired distinctiveness along a category-relevant dimension generalizes to complex object shape and motion.

The global acquired distinctiveness along the relevant dimension replicates several previous results using morphed objects. Goldstone and Steyvers (2001) first observed the effect in a set of faces in which each “dimension” consisted of a morphline between two faces. Subjects categorized faces according to their resemblance to faces along one morphline while ignoring resemblance to faces along the other morphline. This resulted in perceptual gains specific to the relevant morphline (although in this study the gains were measured during a second categorization task rather than a visual discrimination task). This effect was replicated and extended using similar stimulus spaces of objects (Folstein et al., 2012a; van der Linden et al., 2013; Van Gulick and Gauthier, 2014). Related work found acquired distinctiveness in the ventral stream of the visual system: during a task that did not require categorization of the stimuli, pairs of cars that differed along the relevant dimension elicited less fMRI adaptation than pairs of cars differing along the irrelevant dimension (Folstein et al., 2013; but see van der Linden et al., 2013).

We also sought evidence for learned categorical perception. Because past work finding learned categorical perception effects had used multidimensional spaces with simple dimensions or using morphlines without any irrelevant shape variance (Goldstone, 1994; Notman et al., 2005; Wallraven et al., 2014), we hypothesized that it might be easier to observe categorical perception in multidimensional spaces defined by dimensions belonging to separate visual areas—in this case shape and motion. This hypothesis was not confirmed, either because there was no effect or the effect size was much smaller than predicted by Goldstone (1994). This null result is consistent with several recent studies of category learning using morphspaces and continuous shape spaces, none of which found any perceptual advantage along the relevant dimension localized to the category boundary (Op de Beeck et al., 2003; Folstein et al., 2012a; Van Gulick and Gauthier, 2014). Besides the afore mentioned evidence for categorical perception in faces (e.g., Beale and Keil, 1995; Viviani et al., 2007), one particularly relevant counterexample is the study by Wallraven et al. (2014), which reported robust categorical perception when participants learned to categorize a single morphline between two amorphous novel shapes. In that study, there was no irrelevant dimension that had to be ignored—only a single continuum from one shape to the other. In light of the current findings, it is possible that any irrelevant perceptual variance that must be ignored as irrelevant has the effect of reducing learned categorical perception effects.

Our last goal was to test whether category learning caused acquired equivalence in our stimulus space. We found no evidence for acquired equivalence along the irrelevant dimension, nor did we find acquired equivalence between stimuli that differ along the relevant dimension but also share a category. This result is again in line with our recent studies of morphed objects in which we find increases in discriminability for relevant dimensions, but not decreases in discriminability for irrelevant dimensions (Folstein et al., 2012a,b; Van Gulick and Gauthier, 2014). Again, acquired equivalence has been observed for morphed faces (Goldstone et al., 2001; Goldstone and Steyvers, 2001) and for simple separable dimensions (Goldstone, 1994).

Overall, our results support the view that category learning causes stable alterations in visual perception of objects. But importantly, they also suggest that the extremely weak evidence for learned categorical perception and acquired equivalence in non-face objects is not due to interference between relevant and irrelevant shape dimensions. Also, while we have no evidence pertaining to the separability of the dimensions, we speculate based on our results that issues of dimensional separability are also unlikely to account for this pattern. At present, three differences between studies that have and have not observed learned categorical perception seem to us to be worth a closer look. First, learned categorical perception appears quite easy to observe in faces, in contrast to non-face objects. If this could be shown when other stimulus differences were controlled, it might further suggest that objects of face-like expertise are more susceptible to induced categorical perception than other familiar objects, perhaps due to idiosyncrasies of the fusiform face area. Second, and perhaps relatedly, a recent study using only four very highly distinctive non-face objects has reported effects suggestive of categorical perception (Holmes and Wolff, 2012). It is possible that the use of very highly distinctive objects could create separate discrete representations on opposite sides of the category boundary, resulting in categorical perception (Love et al., 2004; Gureckis and Goldstone, 2008). In more highly compressed spaces, unsupervised cluster creation might be less systematic, resulting in clusters near the middle of the space that attract stimuli in both categories. Future studies should examine the effect of highly discriminable “spread out” spaces vs. more commonly used compressed spaces as well as the distribution of stimuli within these spaces. Finally, learned categorical perception has often been observed using morphlines rather than two-dimensional spaces. While this design feature is often confounded with the use of faces (e.g., Beale and Keil, 1995; Viviani et al., 2007), a recent study has observed learned the effect using non-face object stimuli (Wallraven et al., 2014). Thus it is possible that the presence of multiple dimensions could impede learned categorical perception in object stimuli even when dimensions are separable.

### Conflict of interest statement

The authors declare that the research was conducted in the absence of any commercial or financial relationships that could be construed as a potential conflict of interest.

## Footnotes

^1^Some studies have observed dimensional modulation using neural measures during categorization (e.g., Sigala and Logothetis, 2002; Li et al., 2007; van der Linden et al., 2013). Here we emphasize a more stable form of dimensional modulation that is preserved beyond category learning (see also Gauthier and Palmeri, 2002).

^2^While categorical perception may also involve acquired equivalence between same-category objects, increased discriminability along the category boundary can be thought of as a minimal condition for the phenomenon.

^3^This structure was adopted from Op de Beeck et al. (2003). Subjects see stimuli closer to the boundary in the 8 × 8 training space than in the 4 × 4 space. This helps guarantee that the boundary subjects actually learn is between the cross-boundary stimulus pairs on the discrimination test, increasing the probability that we will observe CP.
